# Advanced biorefinery in lower termite-effect of combined pretreatment during the chewing process

**DOI:** 10.1186/1754-6834-5-11

**Published:** 2012-03-05

**Authors:** Jing Ke, Dhrubojyoti D Laskar, Difeng Gao, Shulin Chen

**Affiliations:** 1 Department of Biological Systems Engineering, Washington State University, Pullman, Washington 99164-6120, USA

## Abstract

**Background:**

Currently the major barrier in biomass utilization is the lack of an effective pretreatment of plant cell wall so that the carbohydrates can subsequently be hydrolyzed into sugars for fermentation into fuel or chemical molecules. Termites are highly effective in degrading lignocellulosics and thus can be used as model biological systems for studying plant cell wall degradation.

**Results:**

We discovered a combination of specific structural and compositional modification of the lignin framework and partial degradation of carbohydrates that occurs in softwood with physical chewing by the termite, *Coptotermes formosanus*, which are critical for efficient cell wall digestion. Comparative studies on the termite-chewed and native (control) softwood tissues at the same size were conducted with the aid of advanced analytical techniques such as pyrolysis gas chromatography mass spectrometry, attenuated total reflectance Fourier transform infrared spectroscopy and thermogravimetry. The results strongly suggest a significant increase in the softwood cellulose enzymatic digestibility after termite chewing, accompanied with utilization of holocellulosic counterparts and an increase in the hydrolysable capacity of lignin collectively. In other words, the termite mechanical chewing process combines with specific biological pretreatment on the lignin counterpart in the plant cell wall, resulting in increased enzymatic cellulose digestibility *in vitro*. The specific lignin unlocking mechanism at this chewing stage comprises mainly of the cleavage of specific bonds from the lignin network and the modification and redistribution of functional groups in the resulting chewed plant tissue, which better expose the carbohydrate within the plant cell wall. Moreover, cleavage of the bond between the holocellulosic network and lignin molecule during the chewing process results in much better exposure of the biomass carbohydrate.

**Conclusion:**

Collectively, these data indicate the participation of lignin-related enzyme(s) or polypeptide(s) and/or esterase(s), along with involvement of cellulases and hemicellulases in the chewing process of *C. formosanus*, resulting in an efficient pretreatment of biomass through a combination of mechanical and enzymatic processes. This pretreatment could be mimicked for industrial biomass conversion.

## Background

Transformational scientific knowledge on bioprocessing is required for developing enabling technologies toward converting renewable biomass to targeted biofuels or biochemicals. A major barrier in biomass utilization is the lack of an effective pretreatment of plant cell wall (PCW) so that the carbohydrates can subsequently be hydrolyzed into sugars to be fermented into fuel or chemicals. Existing technologies typically employ thermochemical processes for biomass pretreatment to break down the barriers formed by lignin and lignin-hemicellulose association. Although effective, these processes are energy intensive, environmentally unfavorable and associated with the production of inhibitors that compromise the performance of downstream fermentation. The fact that there is still no successfully demonstrated commercial system in the world for producing biofuels and biochemicals from lignocellulosic biomass underscores the magnitude of the related technical challenges. There is an urgent need for new systems that are more efficient, cost-effective and environmentally-friendly for deconstructing PCW for sugar release.

Nature, through millions of years' evolution, has developed highly efficient biological systems. Wood-feeding termites (Insecta, Isoptera) are among the most effective lignocellulose-recycling invertebrates in terms of the rate and extent of cellulose utilization [1]. Termites work as a complete PCW deconstruction system and are considered to be highly effective for wood degradation as they requires only biomass, moisture and air to function efficiently. Termites' unique mechanisms could serve as an ideal bioconversion model and a novel source of catalysts for refining biofuels and biochemicals. Recently, their ability to convert recalcitrant PCW into a useable energy source of monomer sugars has attracted much interest in the biofuel area [2]. Lower termites are believed to utilize lignocellulosic carbohydrates in a step-wise fashion using cellulases and hemicellulases, with help from lignolytic enzymes [3-6]. However, the pretreatment mechanism of the lignin matrix during the whole digestive process is as of yet unexplored. In addition, the selective modifications to the lignin-hemicellulose matrix for subsequent cellulose hydrolysis have not yet been explored. Lack of such knowledge is a major barrier to the efficient pretreatment of biomass required to enable substantial biomass utilization.

In exploring the mechanisms of PCW degradation by termites, it has always been speculated that the termite chewing action plays an important role in the initial pretreatment. Fujita *et al. *[7] have already demonstrated one of the characteristics of wood degradation by termites to be the mechanical grinding of food by the mandibles to increase the surface area of the substrates. The termite workers chew wood blocks into small particles at an early stage of the digestion process for better access to carbohydrates. During this mechanical grinding of the wood particles, cellulases append to the wood particles or mix with them to initiate the catalysis process [8]. Yoshimura demonstrated that the lower termite *Coptotermes formosanus *crushes and grinds the wood to exhibit sharp edges on the surface, allowing better accessibility by the functional enzymes [3]. It has been reported that termite workers in the Rhinotermitidae family excavate the wood for nesting [9]. When they were moved to a new environment with only wood blocks, they continued their chewing behavior to acquire a large amount of small wood particles for nesting. The nest consisted of ground wood particles pasted together with a special secretion from the salivary glands [9,10]. A large proportion of these particles might be re-digested. During the chewing process, the workers released secretions from their salivary and labial glands for the initial digestion and community activity of feeding stimulation [11].

Termite saliva, which is used for claying of nesting materials, has been reported in previous studies to contain various digestive enzymes. Fujita *et al. *have already cloned two cDNAs that encode premature lysozyme peptides (Rs-Lys1 and Rs-Lys2) from workers of a Japanese damp-wood termite, *Reticulitermes speratus *[12]. Moreover, they demonstrated that the total digestive lysozyme activity is termite in origin and predominates in the salivary glands and, to a minor extent, in the digestive tract. At the same time, Nakashima *et al. *found 80.8% of total cellulase activity stemmed from the salivary glands of *C. formosanus *[13]. Cellobiase is also reported to occur in the salivary glands of *Mastotermes darwiniensis *and *Neotermes koshunensis *[14,15]. It has been proposed that cellulose is first degraded to some extent by carboxymethyl cellulase produced in the salivary glands of termites, and then ingested by protozoa, which finally decompose the cellulose to glucose with their own endo-beta-1,4-glucanases, exo-cellobiohydrolase, and *β*-D-glucosidase [3,16]. For the digestion of hemicelluloses, Inoue *et al. *demonstrated endo-*β*-1,4-xylanase activity in the salivary glands of the lower termite *R. speratus*, however, no *β*-xylosidase activity was documented [17]. For lignin degradation, laccase and phenoloxidase gene expression, as well as benzene-1,2,3-triol oxidation activity, were confirmed in the salivary gland of *R. flavipes *[18].

In termites, the labial glandular ducts open into the oral cavity, from which the watery labial gland secretion is released onto the wood [11]. The labial gland secretion, which contributes to the saliva, is reported to have various species-specific functions in nest construction, as a social nutrient and as a source of digestive enzymes [11,14,19-21]. During communal food exploitation, the labial gland secretion is released onto the food by feeding workers, as demonstrated in the African termite *Schedorhinotermes lamanianus *and the French species *R. santonensis *[22-24], thereby aiding in efficient food exploitation.

It is important to note that current research on lignin modification by termites is mainly focused on the gut process of digestion. Such studies are centered towards the understanding of the symbiont evolution, transcriptomics, meta-transcriptomics and metagenomics of wood-feeding termites, which are necessary for understanding the lignocellulose degradation process. However, there have been no reports nor has attention been drawn towards understanding the combined process of biological lignin modification and physical chewing for cellulose release during the chewing process in termites, which is essential to target the fundamental hurdle in producing cellulosic biofuels-overcoming the barriers of enzymatic hydrolysis of cellulose. In an earlier report, we have already evaluated the possible deconstruction pattern of hardwood lignocellulosics in the clearwing borer system [25]. Here, we expand our investigation of biological lignocellulosic modification during the chewing stage of wood-feeding termites. The purpose of this study is to determine the associated structural changes of the softwood during the mechanical chewing process of the *C. formosanus *termite, and provide new insight into designing new pretreatment processes for biomass conversion. The analysis methods include compositional analysis, enzymatic hydrolysis, pyrolysis gas chromatography mass spectrometry (Py-GC/MS), attenuated total reflectance Fourier transform infrared (ATR-FTIR) spectroscopy and thermogravimetry (TG/DTG) analysis.

## Results and discussion

### Monomer sugar release in termite-chewed softwood

The free monomeric sugars existing in the termite-chewed wood particles, as well as that in the ball milled softwood control, are presented in Table 1. Compared to the artificially ball milled softwood, both the free six carbon (C6) and C5 sugar increased by more than ten times in the chewed softwood, which indicates initial hydrolysis of cellulose and hemicelluloses by termite-originated enzymes. The speculation is that, during the chewing process, termites release digestive enzymes, here including cellulolytic and hemicellulolytic enzymes, onto the chewed particles. A proportion of the sugar is to be further metabolized and the remaining part is liberated from the termite oral cavity with the chewed wood particles for nest construction, together with salivary and/or labial gland secretions. The cellulolytic and hemicellulolytic enzymes attached to the wood particles will continue catalyzing hydrolysis of the cellulose and hemicelluloses, which will cause the free monomer sugars to accumulate in the termite-chewed particles.

**Table 1:** Monomer sugars washed from termite-chewed softwood and undigested control.

Sugar	Undigested softwood (× 10^-8^, g/g)	Termite-chewed softwood (× 10^-8^, g/g)
Fucose	NA	5.35 ± 0.08
Arabinose	25.40 ± 0.6^a^	8.49 ± 0.02
Galactose	1.27 ± 0.01	2.14 ± 0.03
Glucose	15.23 ± 0.07	177.84 ± 1.09
Xylose/Mannose	0.88 ± 0.02	10.17 ± 0.11
Fructose	NA	1.71 ± 0.00
Total	42.78	205.71

^a^Mean of three replicate analyses. NA: not applicable.

### Compositional changes in chewed softwood

For the control and termite-chewed softwood tissues in this study, lignin concentrations were estimated using acetyl bromide (AcBr) extinction coefficients as previously demonstrated [6]. The AcBr analysis determined the lignin content to be 20.83% and 22.81% for control and termite-chewed softwood cell wall residues (CWRs), respectively (Table 2). Interestingly, a slight increase in the lignin content was observed in the softwood tissues after the process of chewing by termites. This increase in the lignin content is possibly due to the relative decrease in the amount of the holocellulosic counterparts in the PCW after grinding by termites, that is, cellulose and/or hemicellulose were preferably degraded and/or utilized by the termites during the chewing process. These findings demonstrate the restricted capacity of lignin degradation during the chewing process of softwood tissues in termites.

**Table 2:** Chemical composition of termite-chewed and control sample.

	Undigested softwood (%)	Termite-chewed softwood (%)
Glucose	36.25 ± 1.16^a^	24.05 ± 0.92
Galactose	6.30 ± 0.18	5.85 ± 0.20
Xylose/Mannose	26.00 ± 0.72	16.99 ± 0.63
Acetyl bromide lignin	20.85 ± 0.14	22.81 ± 0.09
Acid insoluble lignin	18.13 ± 0.82	18.68 ± 0.89
Acid soluble lignin	2.62 ± 0.18	3.78 ± 0.23

^a^Mean of three replicate analyses.

To support such a possibility, chemical compositional analyses on the softwood tissues were carried out to evaluate the relative amounts of acid-soluble and insoluble lignin, together with C6 and C5 carbohydrates, prior to and after the chewing process in termites (Table 2). Thus, the calculated acid-soluble lignin content (expressed as percentage weight of initial biomass) after dilute H_2_SO_4 _acid hydrolysis were 2.62% and 3.78% for the control and termite-chewed softwood tissues, respectively. The 10% increase in lignin content, as observed in the AcBr analysis, correlates with the 44% increase in acid-soluble lignin content, which is speculated to reveal an increase in the hydrolysable capacity of lignin in softwood after the termite chewing process. Meanwhile, the respective decreases of the C6 and C5 sugars in the chewed sample, by 32.8% and 34.7% respectively, supports the previous conclusion that cellulolytic and hemicellulolytic enzymes are released during the termite chewing process, catalyzing the hydrolysis of cellulose and hemicelluloses; the majority of the released sugars are assimilated by the termites.

### Potential change of enzymatic sugar release after termite chewing

**Figure 1:**
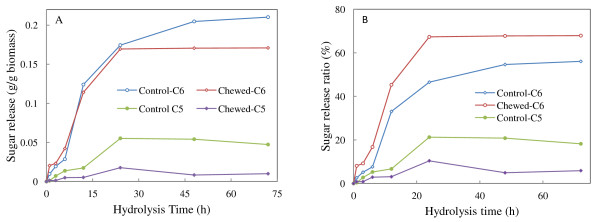
**Influence of termite chewing on enzymatic release of C5 and C6 sugars from the softwood**. **(A) **Amount of sugars released from per gram of softwood biomass. **(B) **Conversion ratio of cellulose and hemicelluloses.

As Figure 1A shows, the chewed sample showed more rapid release of C6 sugars over the first 12 hours of hydrolysis than the control sample. Thus, the termite chewing process can be considered to have a pretreatment effect on the lignin-hemicellulose matrix, exposing the cellulose to enzyme activity to a greater degree than in the ball milled softwood control. Due to the limited cellulose substrate content in the chewed sample (Table 2), the hydrolysis rate slowed after 12 hours of hydrolysis and the enhancement in the release of C6 sugars was not maintained after 24 hours' hydrolysis. Meanwhile, the conversion ratio of cellulose in the termite-chewed softwood significantly increased compared with the control, which demonstrates that termite chewing enhances the release of cellulose. The conversion rates of hemicelluloses to C5 sugars are below 20% for both the termite-chewed and control samples because no specific hemicellulase was used for the hydrolysis. In comparison to the control, the termite-chewed softwood was less susceptible to the release and/or conversion of C5 sugars (Figure 1A, B), which is supported by the lower hemicellulose content (Table 2).

These results strongly indicate that the chewing process of softwood by termites has effectively acted upon the hemicellulosic counterpart and/or lignin-hemicellulose association, preferentially utilizing the C5 sugars by removal and degradation of the hemicellulose framework at the early digestion stage. During the termite chewing process, there are labial and/or salivary secretions, containing cellulases, hemicellulases and lignolytic enzymes, released on not only the chewed and liberated wood particles, but also the wood blocks. This erodes the softwood in a combined mechanical and enzymatic process for efficient initial pretreatment. This initial degradation also causes a more rapid drop in the strength properties of wood, which reflects holocellulose depolymerization.

### Pyrolysis gas chromatography mass spectrometry of monomeric lignin composition changes during the termite chewing process

Pyrolysis produced partial degradation of the lignin side chains, but the aromatic-ring substituent (hydroxyl and methoxyl groups) remained intact, making it possible to identify products arising from the lignin units [26]. Py-GC/MS is a rapid and highly sensitive method for characterizing the chemical structure of lignin, allowing the analysis of a small sample without prior manipulation and isolation [27]. Although there is abundant information on the analytical pyrolysis of different wood types, reports on biologically modified wood are scarce [28,29]. In this study, Py-GC/MS provides qualitative information on *in situ *chemical structural changes of lignin subunits after termite chewing.

**Figure 2:**
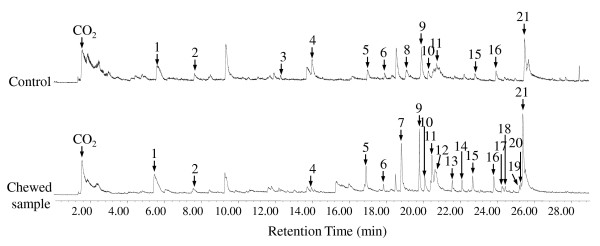
**Pyrogram at 340**°**C**. Py-GC/MS spectra of termite-chewed softwood and undigested control at 340°C. See the details of the labeled pyrolysates in Table 3 and Figure 3.

**Figure 3:**
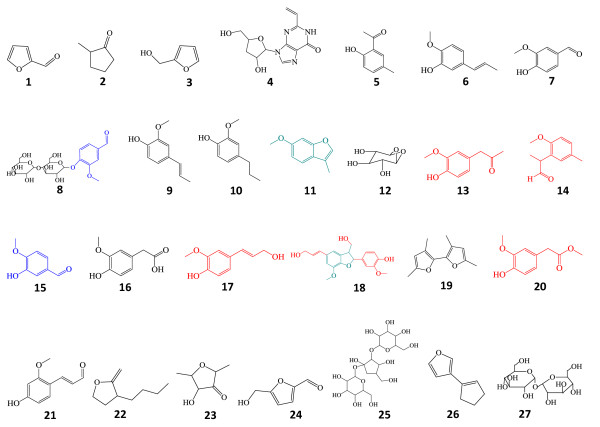
**Compound structures**. Assignment of all the structures of pyrolysates labeled in Figure 2 and 4.

Pyrolysis of the softwood biomass yielded a wide range of products, of which the most characteristic are lignin-related guaiacol derivatives, cellulose-related glucosan derivatives and hemicellulose-related furfural derivatives. In agreement with the information provided by the composition analysis and sugar release ability analysis, the increased lignin content (estimated from the total amount of lignin pyrolysis products) corresponded to the chewed softwood. Figures 2 and 3 and Table 3 demonstrate the compositional changes of the termite-chewed softwood by pyrolysis at 340°C. The related pyrolyzed lignin-derived compounds increased after the termite chewing, indicating initial degradation of lignocellulosic carbohydrates in the termite buccal cavity, as well as no obvious degradation of lignin. Also, the furfural derivatives from the hemicelluloses and glucosan derivatives from the cellulose in the chewed sample were significantly lower than those in the control sample; leaving the relative content of phenols greatly increased in the chewed sample. This specified the metabolism of hemicelluloses and cellulose during oral digestion by termites. Moreover, Py-GC/MS supported that the composition of the lignin-derived fraction from the termite orally digested softwood varied from that of the undigested control.

**Table 3:** Percentages of pyrolysis products from termite-chewed softwood and undigested control at 340°C.

	Name	Molecular formula	Molecular weight	Retention time (min)	*Control (%)	Chewed softwood (%)
**1**	Furfural	C5H4O2	96	5.666	0.37	0.07
**2**	2-methyl-cyclopentanone	C6H10O	98	7.628	0.04	0.01
**3**	2-furanmethanol	C5H6O2	98	12.257	0.03	NA
**4**	2-vinyl-9-[3-deoxy-*β*-d-ribofuranosyl]hypoxanthine	C12H14N4O4	278	14.013	0.10	0.10
**5**	1-(2-hydroxy-5-methylphenyl)-ethanone	C9H10O2	150	16.968	0.24	2.25
**6**	2-methoxy-5-(1-propenyl)-,(E)-phenol	C10H12O2	164	17.917	0.20	0.23
**7**	Vanillin	C8H8O3	152	18.866	NA	4.09
**8**	Vanillin lactoside	C20H28O13	476	19.164	0.23	NA
**9**	2-methoxy-4-(1-propenyl)-(E)-phenol	C10H12O2	164	19.858	1.06	4.12
**10**	2-methoxy-4-propyl-phenol	C10H14O2	166	20.126	0.18	1.83
**11**	6-methoxy-3-methylbenzofuran	C10H10O2	162	20.45	0.33	4.26
**12**	Levoglucosan	C6H10O5	162	20.687	NA	2.08
**13**	1-(4-hydroxy-3-methoxyphenyl)-2-propanone	C10H12O3	180	21.636	NA	0.61
**14**	2-methoxy-*α*,5-dimethyl-benzeneacetaldehyde	C11H14O2	178	22.153	NA	0.93
**15**	3-hydroxy-4-methoxy-benzaldehyde	C8H8O3	152	22.732	0.20	1.27
**16**	4-hydroxy-3-methoxy-benzeneacetic acid	C9H10O4	182	23.866	0.30	1.18
**17**	4-((1E)-3-hydroxy-1-propenyl)-2-methoxyphenol	C10H12O3	180	24.319	NA	0.12
**18**	Methoxyphenyl)-5-(3-hydroxy-1-propenyl)-7- methoxy-2,3-dihydro-2-(4-hydroxy-3-3-benzofuranmethanol	C20H22O6	358	24.487	NA	0.20
**19**	3,3',5,5'-tetramethyl-2,2'-bifuryl	C12H14O2	190	25.251	NA	0.27
**20**	Benzeneacetic acid, 4-hydroxy-3-methoxy-, methyl ester	C10H12O4	196	25.333	NA	0.29
**21**	4-hydroxy-2-methoxycinnamaldehyde	C10H10O3	178	25.436	1.24	7.14

See the structures of the labeled pyrolysates in Figure 3. NA: not applicable

The pyrograms (Figure 2) show a series of products characteristic of pyrolysis of phenylpropanoid compounds in both termite-chewed and native softwood. The main pyrolyzed products in the undigested softwood are simple-structural lignin derivatives (peaks 5, 6, 9, 10, 11, 15, 16, 21), and there are new pyrolyzed lignin derivatives in the preliminarily digested sample, such as compounds **7**, 12, 13, 14, 17, 18, 19, 20. The complex structure (peak 8) in the native softwood sample reveals the linkage of hemicellulose and lignin in the PCW; hence, its disappearance after termite chewing demonstrates termite-induced bond cleavage between holocellulose and lignin, which is supported by the increasing amount of pyrolyzed compound 15 in the chewed sample. The significant increase of the relative amount of pyrolysate 16 in the chewed sample suggests the generation of lignin carbonyl groups by the pretreatment process of termite chewing to decrease the competitive adsorption effect on cellulases, since an increase in the carboxylic content of the lignin preparation is reported to result in an increased yield of cellulose hydrolysis [30]. The newly appearing pyrolyzed compounds (peaks 12 and 19) in the chewed sample at a low pyrolysis temperature indicate depolymerization and degradation of both cellulose and hemicellulose. New compounds of 13, 14, 17, 18, and 20 pyrolyzed from the termite-chewed sample demonstrate the modification of lignin after oral digestion. The appearance of pyrolysate 14 from chewed softwood refers to the dehydroxylation of the phenolic hydroxyl group. This functional group change is considered to remove the inhibitory effect of lignin since phenolic hydroxyl groups have been reported to exhibit critical inhibitory effects on cellulytic enzymes [31]. Compound 18 should be a small fragment pyrolyzed from the lignin molecular with phenylcoumaran (*β*-5') substructure; its appearance only in the chewed sample can be attributed to the specific bond cleavage of the inter-unit linkage of lignin.

**Figure 4:**
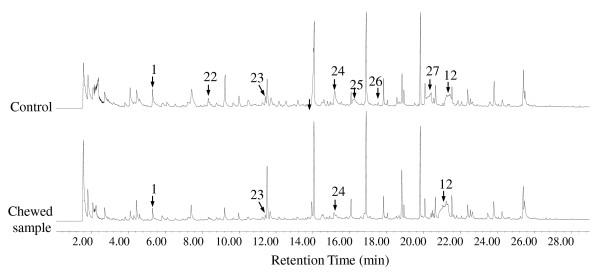
**Pyrogram at 510°C**. Py-GC/MS spectra of termite-chewed softwood and undigested control at 510°C. See the details of the labeled pyrolysates in Table 4 and Figure 3.

Then pyrolysis was performed at a temperature of 510°C (Figures 3, 4 and Table 4) for termite feces and undigested control; the lignin derivatives were released at approximately the same amount for the two samples, indicating no further degradation in the lignin structure by termite. Since a high temperature will be more effective in the cleavage of bonds with high dissociation energy, even if there is lignin modification it is hard to be reflected in the high temperature pyrolysis pyrogram. During high temperature pyrolysis, the cellulose and hemicellulose becomes more easily pyrolyzed into small molecule volatiles; however, there still are some complex structure coming out (peak 25) due to protection by the cell wall lignin. Disappearance of compound 25 in the chewed sample indicates that cleavage occurs between the cellulose and hemicellulose molecules. Disappearance of pyrolytic compounds 22, 26 and 27, as well as the decrease of compounds 23 and 24, in the termite-chewed sample indicate degradation and modification of hemicelluloses and cellulose.

**Table 4:** Percentages of pyrolysates from termite-chewed and control sample at 510°C.

	Name	Molecular formula	Molecular weight	Retention time (min)	Control^a ^(%)	Chewed softwood (%)
**1**	Furfural	C5H4O2	96	5.324	1.21	0.88
**22**	3-butyldihydro-2(3H)-furanone	C8H14O2	142	8.340	0.50	NA
**23**	2,5-dimethyl-4-hydroxy-3(2H)-furanone	C6H8O3	128	11.442	0.57	0.26
**24**	5-(hydroxymethyl)-2-furancarboxaldehyde	C6H6O3	126	15.191	1.96	0.37
**25^b^**	*α*-D-glucopyranoside, O-*α*-D-glucopyranosyl-(1.fwdarw.3)-*β*-D-fructofuranosyl	C18H32O16	504	16.226	1.49	NA
**26^c^**	3-(1-cyclopentenyl) furan	C9H10O	134	17.555	0.12	NA
**27**	*α*-D-glucopyranoside, *α*-D-glucopyranosyl	C12H22O11	342	20.182-20.432	3.02	NA
**12^d^**	Levoglucosan	C6H10O5	162	21.200-21.511	4.33	9.02

^a^Mean of three replicate analyses; ^b^cleavage between cellulose and hemicelluloses molecules; ^c^prefer degradation of hemicelluloses units; ^d^probable depolymerization of the cellulose fiber. See the structures of the labeled pyrolysates in Figure 3.

### Attenuated total reflectance Fourier transform infrared spectroscopic analysis of functional group changes during termite chewing process

**Figure 5:**
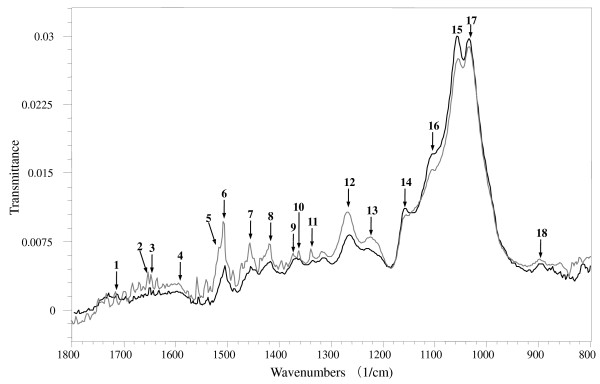
**Spectroscopy**. Selected FTIR spectra, 1800 to 800 cm^-1 ^region, for functional group changes after termite chewing. Black: undigested softwood control; grey: termite-chewed sample. See Table 5 for assignment of FTIR signals.

The FTIR spectra of termite-chewed softwood and the undigested control are shown on Figure 5. Table 5 lists the main assignments of the functional groups in FTIR bands. ATR-FTIR analysis of the lignin from the undigested wood sample showed spectra dominated by aromatic ring vibration and C = O bonds (peaks 4 and 6) [32]. The spectrum of the termite-chewed sample showed higher intensity peaks, indicating the exposure and more vibration of aromatic rings (also supported by decreases in peaks 14 and 17) and the C = O bond (also supported by peaks 1 and 3) [32-34]. The increasing intensity of peak 7 and 10 of the chewed sample can be attributed to the metabolism of -CH_3 _and -CH_2 _groups. It is speculated that the removal of methyl group will help to alleviate the steric hindrance effect of lignin to cellulytic enzymes. In addition, the lignin hydroxyl group was also modified (peak 11), which supports the previous Py-GC/MS data on possible phenolic dehydroxylation (Figures 2 and 4) for the removal of the lignin inhibitory effect. Interestingly, the most obvious change was observed in the range of 1300 to 1600 cm^-1^, indicating greater exposure of the guaiacyl rings and C-O bonds (peak 12), as well as esterification of -OCH_3 _groups (peak 13) [34]. Here, greater exposure of aromatic rings and C-O bonds might be attributed to the lignin side-chain modification; together with phenolic methoxyl group modification, it may help with downstream hydrolysis by alleviating steric hindrance effects of lignin. Meanwhile, the shape change of peak 16 may be attributed to the deformation vibrations of C-O bonds in secondary alcohols and aliphatic ethers, and vibrations of ester linkages on lignin side chains. Increasing intensity of peak 9 and decreasing intensity of peaks 15 and 18 of the chewed sample indicated more bending of aliphatic C-H, as well as less polysaccharide content there (Table 5) [32-34]. Greater intensities of both the peaks 2 and 5 suggest the existence of proteins in the chewed sample, which were assumed to be digestion enzymes from termite saliva and labial gland secretion.

**Table 5:** Main assignments of lignin, polysaccharide and protein Fourier transform infrared bands.^a^

	Wavenumbers (cm^-1^)	Assignments
**Lignin**		
1	1714 to 1725	Stretching of C = O unconjugated to aromatic rings (oxidized side-chains)
3	1655	Stretching of C = O conjugated to aromatic rings
4	1594 to 1609	Aromatic ring vibrations and C = O stretching
6	1504 to 1515	Aromatic ring vibrations
7	1462 to 1464	Asymmetric C-H bending (in CH_3 _and -CH_2_-)
8	1421 to 1424	Aromatic ring vibrations
10	1365	Symmetric deformation of C-H in methyl groups
11	1360	Phenolic hydroxyl vibrations
12	1270 (shoulder)	Vibrations of guaiacyl rings and stretching vibrations of C-O bonds
13	1216 to 1225	C-C, C-O and C = O stretching (G condensed > G etherified)
14	1160	Deformation vibrations of C-H bonds on benzene rings
16	1090 to 1075	Deformation vibrations of C-O bonds in secondary alcohols and aliphatic ethers
17	1030 to 1033	Deformation vibrations of C-H bonds in aromatic rings
**Polysaccharide**		
9	1370	Symmetric bending of aliphatic C-H
15	1030 to 1170	C-O stretching in alcohols
18	890	β-Glycosidic linkages in pyranose units
**Protein**		
2	1655 to 1658	C = O stretching in amides (I)
5	1516	C = O stretching in amides (II)

^a^Referred to references 32 to 34.

In summary, the FTIR data showed that, to alleviate the lignin inhibitory effect, the termite oral digestion might have induced exposure and destruction of the aromatic ring; cleavage of bonds C = O, C-H, O-H and C-O in lignin methoxyl groups, secondary alcohols and aliphatic ethers; deformation of the lignolytic C-H bond in -CH_3 _and -CH_2 _groups; initial degradation of polysaccharide; and secretion of proteins during this process.

### Thermogravimetry analysis for thermal stability of termite-chewed softwood

**Figure 6:**
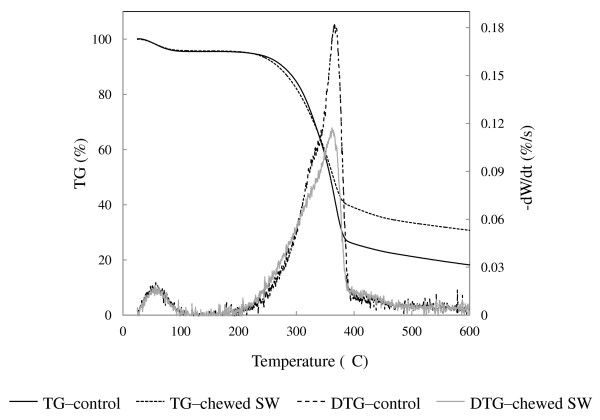
**Thermogravimetry kinetics of thermal decomposition of termite-chewed softwood and undigested control**.

The TG/DTG curves of termite-chewed softwood and native control are shown in Figure 6. This analysis was done to investigate the changes of thermal degradation kinetics of softwood after termite chewing. Wood decomposition commences at around 200°C with the decomposition of lignin and hemicellulose moieties, while the decomposition of the xylan component takes place at around 300°C [35]. The shoulder on the DTG curve of undigested wood represents the xylan present (Figure 6). The highest DTG peak of the undigested wood spectrum at around 360°C corresponds to the decomposition of cellulose [36].

While comparing the thermal decomposition behavior of the termite-chewed softwood to the undigested control, the DTG curve clearly showed large fluctuations during decomposition of the biomass. Chewed biomass decomposition began earlier and the maximum rate shifted left compared to the softwood control, indicating that a lower temperature is capable of converting the chewed sample to volatiles (Figure 6). The lowering of initiation temperature corresponds to a decrease in minimum energy required to start the active gasification reaction in the chewed wood. Meanwhile, the maximum rate of thermal decomposition of the chewed sample largely decreased, showing low cellulose and/or hemicellulose content that also was demonstrated by Py-GC/MS.

Lignin decomposition usually occurs over a wide temperature range, which has pronounced temperature maxima up to 900°C [37,38]. The starting point for termite-chewed softwood on the DTG curve (Figure 6) shifted to the left and increased pyrolysis rate from 200°C to 300°C, thereby indicating a rapid decomposition of the chewed wood particles. Lignin side chain oxidation with production of more carbonyl groups in the chewed sample (Figure 4) might be one of the contributing factors for the initial thermal degradation stage [39], which was also demonstrated by Py-GC/MS. On the other hand, the TG curves showed a larger residual amount of biomass in termite-chewed wood at a high temperature between 400 and 600°C in comparison to the undigested sample. A possible explanation is that once lignin was modified, part of its intermediate fragments will be rearranged through condensation and re-polymerization [40], leading to new structures with more stability. Such rearrangement might either occur during the chewing process in termites or possibly in the pyrolysis process.

By employing the model as described in the method section, the acquired activation energies and pre-exponential factors at different temperature stages will help reveal the chemical structure of biomass components [41]. Table 6 shows that, during the low temperature pyrolysis (220 to 376°C), the activation energy decreased after termite chewing, while the pre-exponential factor increased. This could be attributed to the degradation of carbohydrates and modification of lignin, leading to more exposure and reactivity of the carbohydrate. The participation of more modified thermal-unstable lignin in this pyrolysis step, as indicated by the shift of the DTG-chewed sample curve to a lower temperature in Figure 6, may contribute to the decrease of the activation energy in this temperature range. At a pyrolysis temperature of 376 to 539°C, the activation energy of the softwood biomass significantly increased after termite chewing, indicating that within this temperature range the residual lignin for pyrolysis should have been rearranged and was more stable than the undigested control. This can be explained as a result of lignin functional group modification and/or lignolytic molecules contributing towards increased stabilization of the residual lignin structure by rearrangement. The pre-exponential factor of termite-chewed wood increased at this stage, indicating the necessity of a higher rate of molecular collisions for the rearranged lignin component of the termite digested sample. At the highest temperature (539 to 595°C) pyrolysis stage, the activation energy of the termite-chewed sample decreased, together with an increase of the pre-exponential factor. The decomposition of lignin occurred within a wide temperature range [41], indicating that the remaining lignin structure, which is thermally intractable, was more reactive than the undigested control after termite chewing, which is speculated to be relevant to the cleavage of stable ether bonds (that is, *5*-*5*' [42]) within the lignin network and redistribution of lignin functional groups in the chewed plant tissue. This further supported the previous Py-GC/MS data.

**Table 6:** Kinetic parameters of thermal decomposition of termite-chewed softwood and undigested control at a heating rate of 10°C min^-1 ^in nitrogen atmosphere.

Temperature (°C)	Samples	*E *(kJ mol^-1^)	*A*(s^-1^)	R (Jmol^-1^K^-1^)
220 to 376	Control	48.99	4.09 × 10^4^	0.9647
	Chewed	46.73	4.98 × 10^4^	0.9861
376 to 539	Control	2.76	6.48 × 10^6^	0.9654
	Chewed	6.02	8.68 × 10^6^	0.9908
539 to 595	Control	35.16	7.11 × 10^5^	0.9474
	Chewed	34.67	7.41 × 10^5^	0.9473

*A*: pre-exponential or frequency factor; *E*: activation energy; R: gas constant.

**Figure 7:**
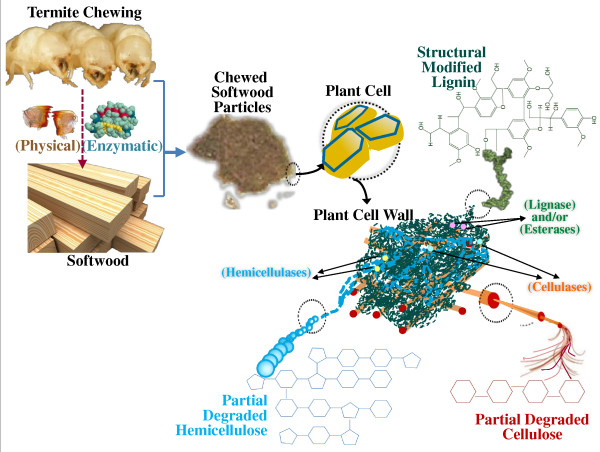
**A proposed biomass destruction process during the termite-chewing process**.

Although the PCW deconstruction process in termite gut is well established [4-6,43], there have been no studies on the initial lignin pretreatment effect during the chewing. Moreover, earlier reports speculate that lignin deconstruction is initiated in the gut; however, they have not identified the specific site(s) [44]. Overall, this study demonstrated an enhanced conversion ratio of cellulose after a combined mechanical and enzymatic pretreatment during termite oral digestion. Associated utilization of holocellulosic counterparts and an increase of hydrolysable lignin in the PCW after termite chewing were also evident. In line with our earlier studies on lignin destruction in the termite gut [5,6], the chewing process contributes to the overall lignin modification by initiating the pretreatment at an early stage for timesaving. Enzymes, such as laccase and phenoloxidase [18], released from the salivary glands and labial glands into the oral cavity during this stage should attach to the lignin substrate and function persistently during the passage of the wood diet through the gut. The main chemical reactions occurring during this stage are dehydroxylation of phenolic hydroxyl groups, side-chain oxidation and *β-*5' substructure modification. These reactions together with additive ring demethoxylation, end-unit cleavage and β-β' substructure modification further happened within the gut for a step-wise lignin disintegrate [5,6]. Results in this study support that modification of lignin structures occurs without degradation in any significant scale during termite chewing. In this regard, due to greater exposure of its polymeric network, modification of lignin are mainly associated with functional group changes and bond cleavage, possibly by depolymerization. These activities indicate the required participation of lignin-related enzyme(s) and/or polypeptide(s) and/or esterase during termite chewing behavior for the initial pretreatment on biomass. Also, the decrease of cellulose and hemicelluloses in the chewed sample infers the involvement of cellulases and hemicellulases in this process. All the results suggest that the PCW deconstruction process starts within the oral cavity of this termite species. The process involves not only mechanical grinding for particle size reduction and amorphous cellulose breakdown but also selective biochemical pretreatment of lignin, which makes this process more unusual in the approach to pretreatment of biomass (Figure 7). This reveals the advantages of the *C. formosanus *termite over white-rot fungi and other lignocellulosic-degrading microbes, in that it works as a highly efficient continuous and well-integrated system for lignin-hemicellulose matrix deconstruction. Not all the lower termite species have this pre-digestion process; it is absent in several termite species [45]. This efficient wood degrader provides us with new information for improving pretreatment of lignocellulosics by providing the scientific base for integrating mechanical grinding and enzymatic lignin modification.

## Conclusions

The termite chewing process induced selective pretreatment on the lignin counterpart and/or lignin-hemicellulose association. Consequently, the structural modification within the lignin matrix during the chewing process resulted in better exposure of the carbohydrate component within the PCW. These results could significantly contribute towards the current understanding of specific unlocking modification on lignin structure by the lower termites for efficient cellulose utilization, as well as promoting the existing biomass pretreatment technology for effective disintegration of the PCW structure to produce bioenergy-derived products and fuels. Combining the achievements of nature with those of mankind should lead to new breakthroughs in science and technology that are critical for converting lignocelluloses to biofuels.

## Methods

### Termite cultivation and sample preparation

Five hundred *C. formosanus *termite workers, which are responsible for feeding in the colony, were collected in Poplarville, Mississippi, and starved at 28°C and 90% humidity for 40 hours to try to evacuate their alimentary canals. Termite digestion is thought to be accomplished within 24 hours [46-48], and the gut evacuation was supported by the Py-GC/MS results in Additional file 1 with no evidence of lignolytic, cellulosic or hemicellulosic pyrolysate in the pyrogram, thus no feces should have been contained in the chewed sample collected.

The termites were provided with 6.8 × 1.5 × 0.5 inch blocks of Southern pine (*Pinus australis *F. Michx) and left for 15 hours at 28°C and 90% humidity. Termite-chewed softwood particles were collected from the corner of the container, avoiding those in contact with the wood blocks. The lignin in this species of pine consists almost exclusively of guaiacyl propane subunits [49]. The experimental conditions (40 hours of starvation followed by 15 hours of wood-feeding) were repeated several times for different groups of termite workers for sample collection. The particle size of the chewed softwood was approximately 50 μm as estimated under the microscope. The undigested softwood tissues were ball milled to the same particle size, and used as control samples. The effect of this starvation treatment on enzyme secretion of termite workers was ignored since short-term starvation, and even the long-term starvation, does not lead to complete loss of enzyme secretion [50,51], and wood provision after 40 hours will help termite workers re-acquire their normal level of enzyme production [42]. However, we need keep in mind of the probable lowered secretion of cellulases and lignases from the salivary glands.

### Washing-up of termite-chewed particles for free sugars

The termite-chewed softwood particles, in 100-mg batches, and the same weight of control softwood were individually soaked in 400-μL deionized water and stirred for 6 hours at room temperature. Sugar concentrations in the resulting extracts were determined by the Dionex ion chromatograph (IC). The extractives were filtered through a 0.45-mm filter prior to sample analysis. Sugars were separated on CarboPac PA 20 Guard (4 × 50 mm) and analytical columns (4 × 250 mm) at room temperature (25°C). Sugar detection was by an ED40 electrochemical detector (Dionext Corp., Bannockburn, IL, USA). An AS40 sampler (Dionext Corp.) was used for continuous running and Dionex PeakNet 5.1 chromatography software was used for data analysis.

### Wood composition analysis

For analysis of the acetyl bromide lignin content, both the control and termite-chewed softwood tissues (200 mg) were individually frozen in liquid nitrogen. Extractive-free freeze-dried CWRs were prepared as described previously [25] and then subjected to acetyl bromide analysis [52,53]. The lignin contents of extractive-free CWRs samples for both the control and termite-chewed softwood tissues were estimated in terms of percentage weight of the extractive-free freeze-dried CWRs.

For a further chemical composition analysis, 1 mL 72% sulfuric acid was added into 100 mg of each freeze-dried extractive-free sample separately and incubated in a 30°C water bath for 1 hour. Then, 29 mL deionized water was added into each sample separately, and the two samples were placed in an autoclave (Brinkmann 2540 M, Tuttnauer USA CO. Ltd, Hauppauge, NY, USA) at 121°C for 1 hour, then the water loss was supplemented. After centrifugation, the supernatant was diluted and subjected to the Dionex IC and UV spectroscopy with absorbance at 205 nm (0.2 to 0.7) with an extinction coefficient of 110 L g^-1 ^cm^-1 ^for analysis of contents of sugars and acid soluble lignin [54]. The solid phase was washed three times with deionized water and finally freeze-dried to obtain the acid insoluble lignin.

### Enzymatic hydrolysis of the termite-chewed wood particles

Enzymatic hydrolysis of both the washed termite-chewed and control samples was carried out at 2% of solid component (w/v) in 10 mL sodium citrate buffer using a rotary shaker at 150 rpm. The pH and temperature were adjusted to 4.8 and 50°C, respectively. A mixture of Celluclast 1.5 L (Sigma-Aldrich, Inc., St. Louis, MO, USA) and Novozyme 188 (Sigma-Aldrich, Inc.) with activity loadings of 60 filter paper units and 64 cellobiase units, respectively, as well as 3.2 × 10^-4 ^g sodium azide was used for enzymatic hydrolysis per one gram of substrate. Reaction mixtures were pre-incubated for 30 min prior to the addition of enzymes. Hydrolysates were sampled periodically (1, 3, 6, 12, 24, 48, 72 h) for sugar analysis. Each data point was averaged from three replicates.

Sugar concentrations in the hydrolysates were determined with IC. The hydrolysates were filtered through a 0.45 mm filter prior to sample analysis. Sugars were separated on CarboPac PA 20 Guard (4 × 50 mm) and analytical columns (4 × 250 mm) at room temperature (25°C), and detected by an ED40 electrochemical detector. An AS40 sampler was used for continuous running and Dionex PeakNet 5.1 chromatography software was used for data analysis.

### Pyrolysis gas chromatography mass spectrometry analysis at 340°C and 510°C

Samples were frozen in liquid nitrogen to quench lignin digestion, and then loaded directly into a quartz tube (sample tube). For each sample tube, there was an approximately 10 mg sample. The pyrolysis processes were performed with a CDS 5000 pyrolysis autosampler (Analytical, Inc., Oxford, PA, USA) attached to a Thermo Trace GC 6890N/MSD 5975B gas chromatography mass spectrometry system (Agilent Technologies, Inc., Bellevue, WA, USA). The wood samples were first pretreated at 210°C for 3 min, and then pyrolyzed at a temperature of 340°C and 510°C for 1 min, respectively. The sample of two guts of starved termites was first pretreated at 210°C for 3 min, and then directly pyrolyzed at a temperature of 510°C for 1 min. Finally the volatile products were held in the pyrolysis zone for 56 min, separated on a 30 m × 0.25 μm inner diameter (5% phenyl)-methylpolysiloxane column with helium 4.6 as carrier gas (17.3 mL min^-1^) and identified by interpretation of their electron impact mass spectra in comparison to a NIST Mass Spectral Search 2.0 electronic library. The pyrolysis interface was kept at 210°C and the GC/MS interface at 280°C; the GC/MS was programmed from 40°C (1 min) to 280°C (15 min) at a rate of 6°C min^-1^. The mass spectrometer was operated in electron impact mode (70 eV) at a source temperature of 230°C.

### Attenuated total reflectance Fourier transform infrared spectroscopic analysis

To directly correlate the modification of lignin functional groups with the termite chewing process, ATR-FTIR spectra (4500 to 800 cm^-1^) of both the termite-chewed and undigested softwood particles were obtained with a SHIMADZU IRPrestige-21 Fourier transform infrared Spectrophotometer (Shimadzu Corp., Kyoto, Japan) using approximately 2 mg of each sample. A MIRacle ATR accessory with a high-pressure clamp (PIKE Technologies, Madison, WI, USA) was used. Spectra were obtained using the triangular apodization, a resolution of 4 cm^-1 ^and an interval of 1 cm^-1^. Sixty-four scans were conducted for each background and sample spectra. Baseline and ATR corrections for penetration depth and frequency variations were applied using the Shimadzu IR solution 1.30 software supplied with the equipment.

### Thermogravimetric analysis

TG/DTG analysis is based on the precise study of weight loss during programmed exposure to over a range of temperatures, to determine termite-induced changes in the general characteristics of lignocellulose decomposition and activation energies for bond cleavage under pyrolysis and combustion. It was conducted using a Mettler-Toledo TGA/SDTA851^e ^(Mettler-Toledo, Inc., Columbus, OH, USA). An approximately 5 mg sample was loaded into an alumina pan and vaporized (from 25°C to 600°C, at a heating rate of 10°C min^-1^) under a nitrogen atmosphere with a flow rate of 20 mL min^-1 ^(standard temperature and pressure). A heating rate of 10°C min^-1 ^was selected for the termite-chewed softwood and undigested softwood control.

### Mathematical analysis of thermodegradation

A model consisting of two independent simultaneous reactions was employed to describe the thermodegradation kinetic changes of termite-chewed softwood using the integral method of Coats and Redfern [55]. In this model, each reaction stage was assumed to be first order in the formulation of the kinetic model. The integral equation is cited in Yang *et al. *[56] as follows:

$$\ln\left[\frac{-\ln\left(1-\alpha \right)}{T^{2}}\right]=\ln\left(\frac{A\text{R}}{\beta E}\right)-\frac{E}{\text{R}T}$$

for which, $\alpha =\frac{w_{0}-w}{w_{0}-{\text{w}}_{\text{f}}}$, and $\beta =\frac{\text{d}T}{\text{d}t}=constant$ and where, *T *is the absolute temperature (K), *A *is the pre-exponential or frequency factor (s^-1^), R is the gas constant (Jmol^-1^K^-1^), *E *is the activation energy (Jmol^-1^), *w *is the mass fraction present at any time, w_0 _is the initial mass fraction, and w_f _is the mass fraction at infinity.

Plotting ln[g(α)/T^2^] versus 1/T results in a straight line with a slope of -E/R and a y intercept of ln[(AR)/(βE)], providing the values of E and A.

## Competing interests

The authors declare that they have no competing interests.

## Authors' contributions

JK and DL conceived and designed the experiments. JK and DG performed the experiments. JK and DL analyzed the data. SC contributed reagents, materials and analysis tools. JK and DL wrote the paper. SC mentored and revised the paper. All authors read and approved the final manuscript.

## Supplementary Material

Additional file 1 **Pyrogram**. Py-GC/MS spectrum of the whole guts of two termites starved for 40 hours. The appeared peaks represent CO_2 _and fatty acids, respectively. Non-existence of pyrolysate from lignin, cellulose or hemicellulose suggested complete evacuation of the gut contents. Click here for file
