# Supplementary figures and images for: Advanced biorefinery in lower termite-effect of combined pretreatment during the chewing process
Source: Biotechnol Biofuels. 2012 Mar 5;5:11. doi: 10.1186/1754-6834-5-11 (PMC3310848; doi:10.1186/1754-6834-5-11)

## Slide 1
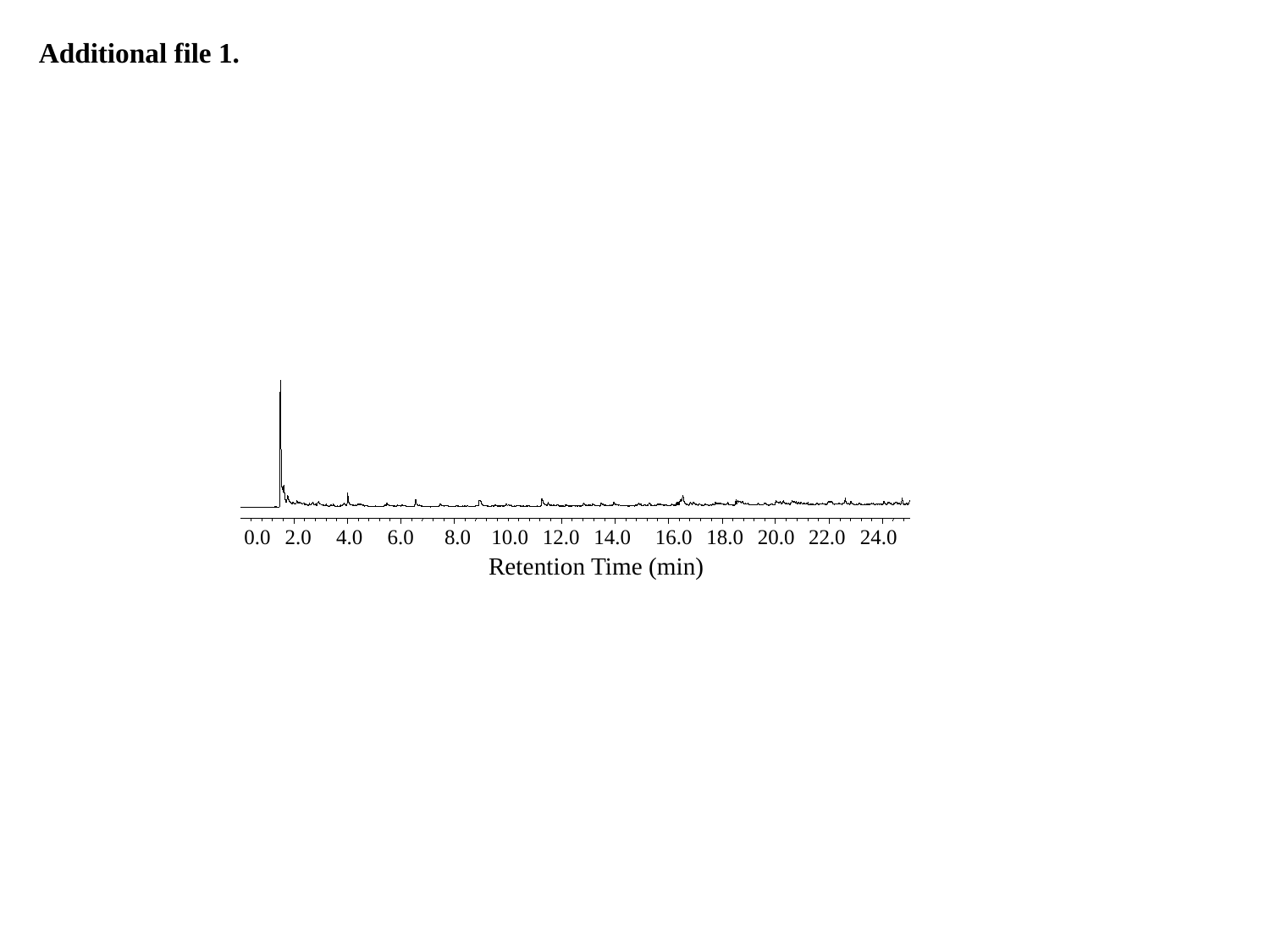

Additional file 1.
0.0
2.0
4.0
6.0
8.0
10.0
12.0
14.0
16.0
18.0
20.0
22.0
24.0
Retention Time (min)

Supplement: Additional file 1 — Pyrogram. Py-GC/MS spectrum of the whole guts of two termites starved for 40 hours. The appeared peaks represent CO2 and fatty acids, respectively. Non-existence of pyrolysate from lignin, cellulose or hemicellulose suggested complete evacuation of the gut contents. [file 1754-6834-5-11-S1.PPT]
